# Development of a Novel Marine-Derived Tricomposite Biomaterial for Bone Regeneration

**DOI:** 10.3390/md21090473

**Published:** 2023-08-28

**Authors:** Bilal Aslam, Aleksandra Augustyniak, Susan A. Clarke, Helena McMahon

**Affiliations:** 1 Circular Bioeconomy Research Group (CIRCBIO), Shannon Applied Biotechnology Centre, Munster Technology University, V92CX88 Tralee, Ireland; bilal1@live.ie (B.A.); aleksandra.augustyniak@mtu.ie (A.A.); 2 School of Nursing and Midwifery, Medical Biology Centre, Queen’s University of Belfast, Belfast BT9 7BL, UK; s.a.clarke@qub.ac.uk

**Keywords:** chitosan, hydroxyapatite, fucoidan, scaffolds, bone tissue engineering

## Abstract

Bone tissue engineering is a promising treatment for bone loss that requires a combination of porous scaffold and osteogenic cells. The aim of this study was to evaluate and develop a tricomposite, biomimetic scaffold consisting of marine-derived biomaterials, namely, chitosan and fucoidan with hydroxyapatite (HA). The effects of chitosan, fucoidan and HA individually and in combination on the proliferation and differentiation of human mesenchymal stem cells (MSCs) were investigated. According to the SEM results, the tricomposite scaffold had a uniform porous structure, which is a key requirement for cell migration, proliferation and vascularisation. The presence of HA and fucoidan in the chitosan tricomposite scaffold was confirmed using FTIR, which showed a slight decrease in porosity and an increase in the density of the tricomposite scaffold compared to other formulations. Fucoidan was found to inhibit cell proliferation at higher concentrations and at earlier time points when applied as a single treatment, but this effect was lost at later time points. Similar results were observed with HA alone. However, both HA and fucoidan increased MSC mineralisation as measured by calcium deposition. Differentiation was significantly enhanced in MSCs cultured on the tricomposite, with increased alkaline phosphatase activity on days 17 and 25. In conclusion, the tricomposite is biocompatible, promotes osteogenesis, and has the structural and compositional properties required of a scaffold for bone tissue engineering. This biomaterial could provide an effective treatment for small bone defects as an alternative to autografts or be the basis for cell attachment and differentiation in ex vivo bone tissue engineering.

## 1. Introduction

Bone injuries and defects present a significant clinical problem. Most are due to trauma and are easily resolved. However, treatment of complex breaks and disease-associated bone pathologies is frequently challenging. Severe skeletal injuries consisting of fractures with a compromised blood supply are often associated with complications, such as mal-union and non-union, which prevent the bone healing naturally [1]. In order for these injuries to heal, both bone regeneration and restoration of blood flow are required, with the current gold standard of treatment involving invasive surgeries [2,3]. Consequently, the requirement for new bone tissues to restore the function of damaged or lost bone is a major clinical need [4]. Bone regeneration strategies have evolved significantly from cellular therapy (delivery of stem cells alone) [2,5] to utilisation of scaffolds (organic and inorganic) [6,7,8,9], hydrogels [10,11,12] and nanofibers [6,13,14,15]. Treating bone defects using scaffolds can be carried out using natural or synthetic bone grafts. Depending on the origin, there are three types of natural grafts, including autografts (transferred from the same individual), allografts (transferred from another person) and xenografts (derived from other species) [16,17,18]. As allografts and xenografts undergo acellularisation process in order to minimise the possibility of an immunological response, they can be categorised as tissue engineering [18]. Synthetic bone grafts use artificially engineered biomaterials to replace bone tissue. They can be classified, according to their composition, into metals, polymers, ceramics or composites [19].

Bone formation is a highly complex process in which various growth mediators and cells provide the cellular signalling for migration, proliferation and differentiation of MSCs into osteogenic cells, thereby driving new tissue synthesis and the associated de- and re-mineralisation processes. Bone engineering strategies aim to mimic these regenerative processes using exogenous or endogenous growth factors with scaffolds. The efficacy of a bone scaffold is dependent upon its ability to support bone cell growth (osteoconductivity). To achieve this, it must mimic the properties of bone extracellular matrix (ECM) as much as possible. Bone ECM comprises organic (30%) and inorganic (70%) components. The organic component is composed of a range of macromolecules, proteins, polysaccharide proteoglycans, glycosaminoglycans, glycoproteins, as well as osteonectin, osteocalcin and collagen fibres, which are predominantly type I. The 70% inorganic component is hydroxyapatite. Scaffolds must also promote osteogenic differentiation (osteoinductivity), which is achieved through the architecture of the scaffold and the inclusion of growth factors. Finally, scaffolds must be biocompatible (supportive of cell attachment and proliferation, along with lack of toxicity and inflammatory reactions) and bioresorbable [8,11,20,21,22,23].

Integration of multiple stimuli in scaffolds, including porosity, pore-size distribution, topography, stiffness (physical) and growth factors, that are similar to natural bone tissue will improve scaffold efficacy [24,25].

Chitosan is a natural cationic polymer with structural similarities to hyaluronic acid of the ECM [26]. It is a linear, semicrystalline polysaccharide composed of (1,4)-2-acetamido-2-deoxy-b-D-glucan (N-acetyl D-glucosamine) and (1,4)-2-amino-2-deoxyb- D-glucan (D-glucosamine) units. It is non-toxic, antibacterial, biodegradable and biocompatible. It is widely used in biomedical applications such as tissue engineering scaffolds, drug delivery, wound dressings, separation membranes and antibacterial coatings [27,28]. Chitosan has also been extensively applied in bone tissue engineering and has been shown to promote cell growth and mineral-rich matrix deposition by osteoblasts with increased osteogenesis in vitro and in vivo [26,29,30,31,32,33,34,35,36]. It is fully resorbable in vivo, where the rate of degradation is related to the molecular mass of chitosan and biocompatibility with physiological medium.

The mineral component of bone is predominantly hydroxyapatite (HA), and its inclusion in bone bioengineering has been extensively reported, with clear evidence that HA scaffolds promote bone formation [6,7,14,32,33,37,38]. Chitosan-based HA composites have also been widely used in reconstruction of bone defects, with evidence of biocompatibility and osteoconductive characteristics [33,39,40]. However, there is very limited evidence that these composites have osteoinductive properties.

To address this, it may be useful to add a third component to composite materials. Fucoidan is a sulphated polyfucose polysaccharide, which is produced and isolated from phaeophyta (brown seaweeds) [41]. As a heparinoid compound, fucoidan mimics the biological activities of heparin, with the capacity to both sequester and potentiate activities of fibroblast growth factors (FGFs), which are involved in tissue repair and angiogenesis [42,43,44]. Therefore, fucoidan could potentially address the need for growth factors in the classic triad of bone tissue engineering. Furthermore, fucoidan is reported to promote osteogenic differentiation, alkaline phosphatase (ALP) levels, type-1 collagen expression, osteocalcin, BMP-2 and increased mineral deposition associated with bone mineralisation [39,45,46,47,48,49].

Based on applications in bone tissue engineering, chitosan and HA are abundantly used in bone scaffold preparation and fucoidan has also been employed for tissue regeneration, but the unique combination of chitosan, fucoidan and HA into one scaffold assembly has not been reported. This research aims to develop such a tricomposite and evaluate its biocompatibility and osteogenic capacity.

## 2. Results

### 2.1. Scaffold Characterisation

#### 2.1.1. Scanning Electron Microscopy (SEM)

**Figure 1:**
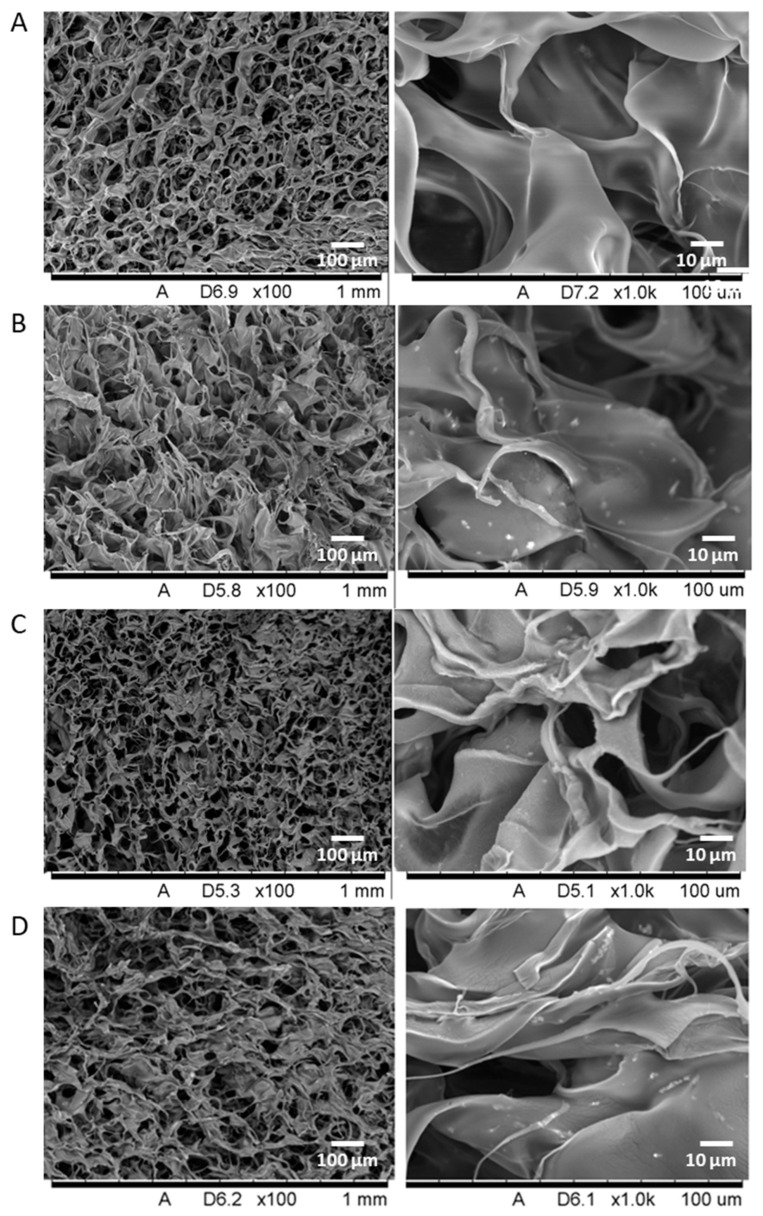
SEM image of bicomposite and tricomposite bioscaffolds developed via the polyblend method. The images capture scaffold development, progressing from 1% chitosan scaffold only (**A**) to chitosan-and-fucoidan bicomposite (1% chitosan: 12.5 µg/mL fucoidan; (**B**)), chitosan-and-hydroxyapatite bicomposite (1% chitosan: 200 µg/mL hydroxyapatite; (**C**)), and tricomposite biomaterial (1% chitosan: 12.5 µg/mL fucoidan: 200 µg/mL hydroxyapatite; (**D**)).

The SEM results (Figure 1) show that the 1% chitosan scaffold has a highly porous structure with a uniform pore-size distribution and a high surface area for cell adhesion and proliferation. Analysis of the chitosan/HA bicomposite material (Figure 1C) shows that HA nanoparticles, which are clearly visible, are uniformly distributed throughout the scaffold. Similarly, the tricomposite (Figure 1D) has a uniform, highly porous structure with embedded HA nanoparticles, and fucoidan appears as a thread-like structure that adheres to the scaffold.

#### 2.1.2. Fourier-Transform Infrared Spectroscopy (FTIR) Analysis

**Figure 2:**
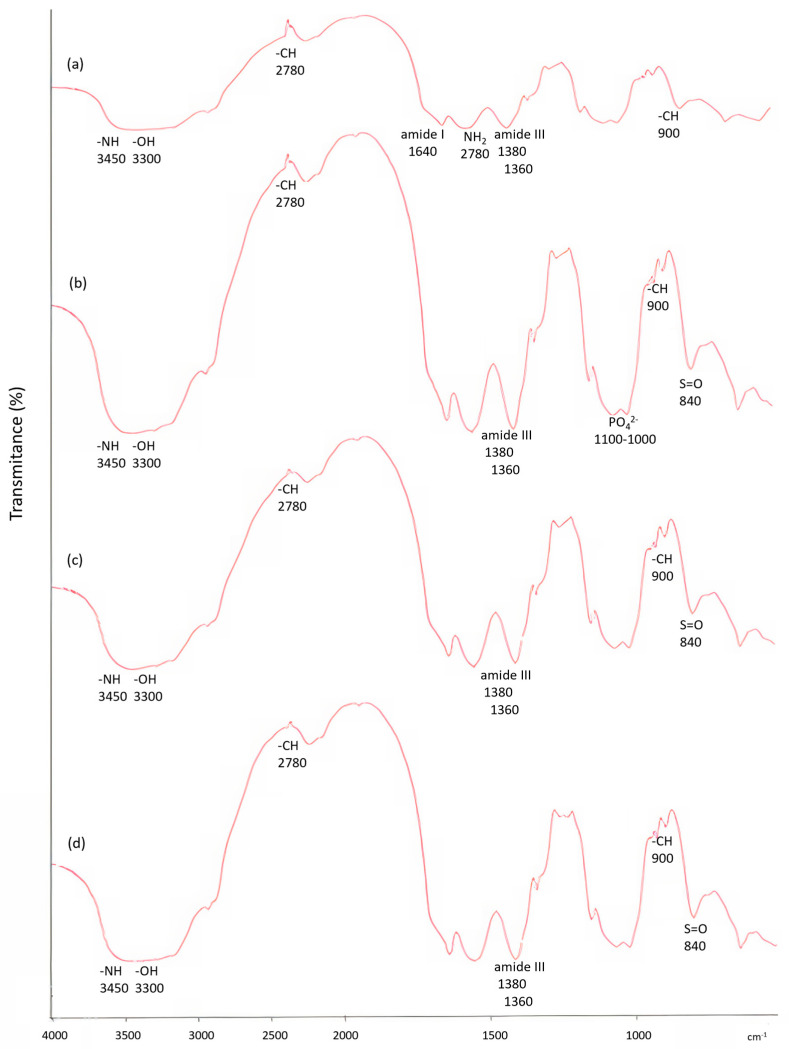
FTIR analysis to confirm presence of hydroxyapatite and fucoidan in the bi- and tricomposite bioscaffolds. Specifically, the spectra are chitosan only (**a**), chitosan/HA bicomposite (**b**), chitosan/fucoidan bicomposite (**c**), and chitosan/fucoidan/HA tricomposite (**d**).

Further confirmation of this was observed using FT-IR (Figure 2). The chitosan spectrum (Figure 2a) shows characteristic peaks at 3450 and 3300 cm^−1^, which correspond to –OH and –NH stretching. In addition, a strong intense peak was observed at 2780 cm^−1^, which corresponds to –CH stretching and is specific to chitosan only. The absorption band of (amide I), –NH_2_ and (amide III) bending are shown at 1640 cm^−1^, 1575 cm^−1^ and 1380 cm^−1^, respectively. Further –CH stretching of glycoside linkage was observed at 900 cm^−1^. The chitosan/HA spectrum (Figure 2b) shows overlapping of chitosan with HA at –OH and –NH at 3450 cm^−1^. It is assumed that the overlapping of –PO_4_^−3^ at 1100–1000 cm^−1^ has occurred. In contrast, in the spectrum of chitosan/fucoidan bicomposite (Figure 2c), there is superposition of the hydroxyl group of chitosan with fucoidan at 3400 cm^−1^, although –CH stretching at 2870 cm^−1^, which is specific to chitosan only, is still visible. Furthermore, fucoidan exhibits its characteristic band at 840 cm^−1^, which is assigned to the sulphate group (S=O) [50,51]. The spectrum of chitosan/fucoidan/HA tricomposite (Figure 2d) shows the main characteristic peaks of fucoidan and HA that are superimposed on chitosan. The characteristic peaks of chitosan were observed at 3450–3300 cm^−1^ for –OH and –NH stretching, respectively. –CH stretching of chitosan was also observed at 2870 cm^−1^. Furthermore, it can be assumed that there is an overlapping of –PO_4_^−3^ from HA and sulphate group from fucoidan at 840 cm^−1^.

#### 2.1.3. Porosity and Density

Porosities of more than 90% are required for tissue engineering applications to facilitate cell migration, growth and vascularisation [52,53]. An ethyl alcohol liquid displacement method was used to evaluate the porosity and density of the bioscaffolds. The results (Table 1) show that the porosity of the chitosan/HA (77.58%) bicomposite is lower than the chitosan scaffold (93.75%), chitosan/fucoidan bicomposite (89.74%) and chitosan/fucoidan/HA tricomposite (92.05%). There is a concomitant increase in density of the HA-containing scaffolds compared to chitosan alone (Table 1). The swelling ratio was calculated to assess the water absorption capacities of the scaffolds and revealed that chitosan scaffolds retained water at 2 h, but there was no further increase observed thereafter up to 96 h (Table 1).

### 2.2. In Vitro Mesenchymal Stem Cell Studies

#### Biocompatibility: Chitosan, Fucoidan and Hydroxyapatite

**Figure 3:**
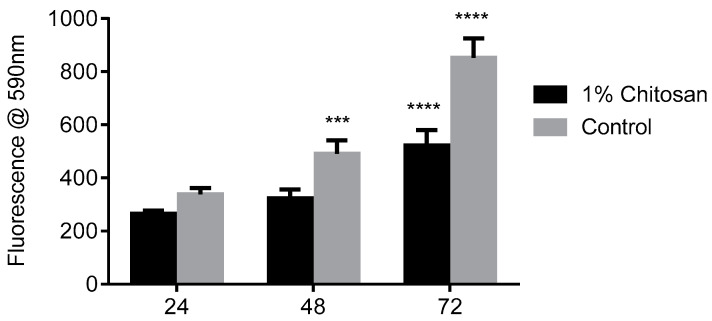
Evaluation of chitosan MSC biocompatibility. MSCs grown without scaffold treated as a control. Viability was measured using the resazurin fluorescence assay, with excitation and emission wavelength of 530 nm of 590 nm. Results are expressed as mean ± S.D, (*n* = 3), (*p* < 0.05); *p*-value (***, 0.001; ****, <0.0001).

The biocompatibility of all three proposed bioscaffold components was evaluated in vitro. Chitosan was found to reduce cell proliferation compared to control cells grown in media only; however, cells continued to proliferate for the duration of the assay and cell viability was above 90%, indicating that chitosan is biocompatible and supports hMSC growth (Figure 3).

**Figure 4:**
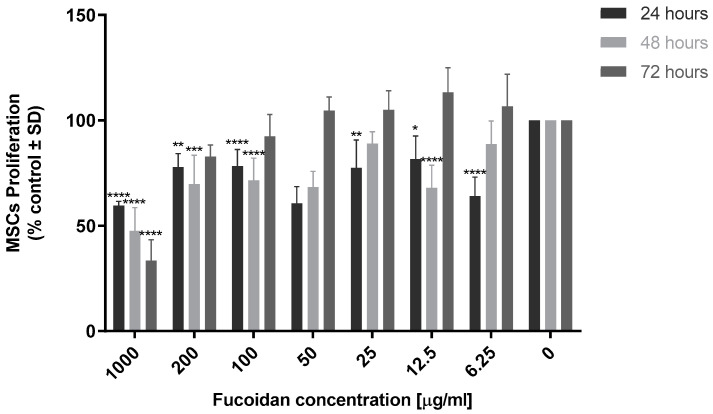
Evaluation of fucoidan MSC biocompatibility. In total, 1 × 10^4^ MSCs were seeded per well on a 96-well plate; following an attachment period, cells were cultured the presence of fucoidan (0 to 1000 µg/mL). Viability was measured at 24, 48 and 72 h using resazurin fluorescence assay, with excitation and emission wavelength of 530 nm and 590 nm. Results are expressed as mean ± S.D (*p* < 0.05), *p*-value (* 0.05, ** 0.01, *** 0.001, **** < 0.0001), (*n* = 3).

Fucoidan MSC cytotoxicity (0 to 1000 µg/mL) was evaluated (Figure 4). Fucoidan was cytotoxic to MSCs at 1000 µg/mL, showing a statistically significant reduction in cell number that increased with time (24–72 h) (*p* < 0.0001). Similarly, at 24 h, all other concentrations of fucoidan reduced cell number compared to the control. However, at concentrations lower than 200 µg/mL, inhibition was reversed by 72 h with no significant difference in cell number compared to the control. Based on these results, concentrations ranging from 6.25 µg/mL to 25 µg/mL were selected for bi- and tricomposite studies.

**Figure 5:**
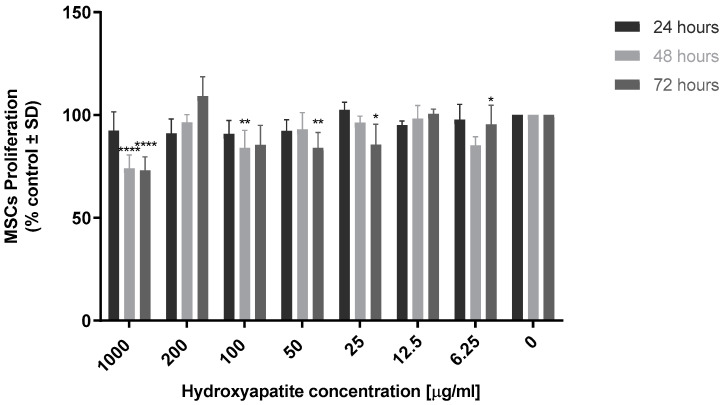
Evaluation of hydroxyapatite MSC biocompatibility. In total, 1 × 10^4^ MSCs were seeded per well on a 96-well plate. Following an attachment period, cells were cultured in the presence of hydroxyapatite (0 to 1000 µg/mL). Viability was measured at 24, 48 and 72 h using resazurin fluorescence assay, with excitation and emission wavelength of 530 nm and 590 nm. Results are expressed as mean ± S.D (*p* < 0.05), *p*-value (* 0.05, ** 0.01, *** 0.001, **** < 0.0001), (*n* = 3).

HA MSC cytotoxicity (0 to 1000 µg/mL) was evaluated (Figure 5). The results showed that cell proliferation at 48 and 72 h was significantly reduced compared to the control (*p* < 0.0001) at a concentration of 1000 µg/mL. Treatment of MSCs with HA at 200 µg/mL stimulated an elevated rate of MSC proliferation at 72 h. HA at lower concentrations had limited effects on cell proliferation, and HA was found to be extremely biocompatible. Based on these results, an HA concentration of 200 µg/mL was selected for bi- and tricomposite studies.

**Figure 6:**
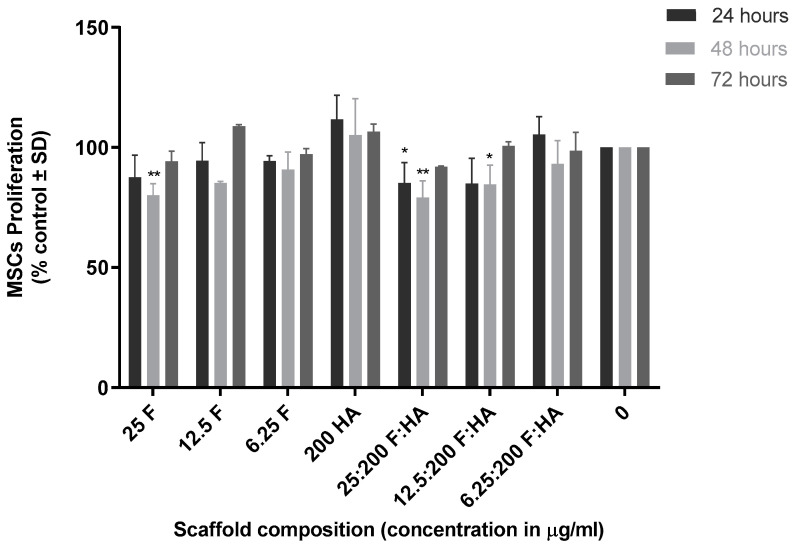
Evaluation of fucoidan/hydroxyapatite co-treatment on MSC proliferation. MSCs were co-treated with fucoidan (F) and hydroxyapatite (HA) at concentrations of 6.25 µg/mL and 12.5 µg/mL, and 25 µg/mL and 200 µg/mL, respectively. Viability was measured using resazurin fluorescence assay, with excitation and emission wavelength of 530 nm and 590 nm, at 24, 48 and 72 h. Results are expressed as mean ± S.D (*p* < 0.05), *p*-value (* 0.05, ** 0.01), (fucoidan, *n* = 3; hydroxyapatite, *n* = 6; and control, *n* = 6) (*p* < 0.05).

To determine if co-exposure of MSCs to both fucoidan and HA had any impact on MSC cell growth, cell proliferation assays were carried out. MSCs were co-treated with fucoidan and HA at concentrations of 6.25 µg/mL and 12.5 µg/mL, and 25 µg/mL and 200 µg/mL, respectively. The overall trend of the results suggested that the presence of fucoidan reduced proliferation at 24 and 48 h, but cell number recovered to the control levels by 72 h (Figure 6). A concentration of 200 µg/mL of HA resulted in slight but non-significant increased cell proliferation and reduced the inhibitory effect of fucoidan at early time points when both were given in combination. Based on these results, 1% chitosan, HA at 200 µg/mL, and 12.5 µg/mL and 25 µg/mL of fucoidan were selected for 3D scaffold preparation and evaluation.

**Figure 7:**
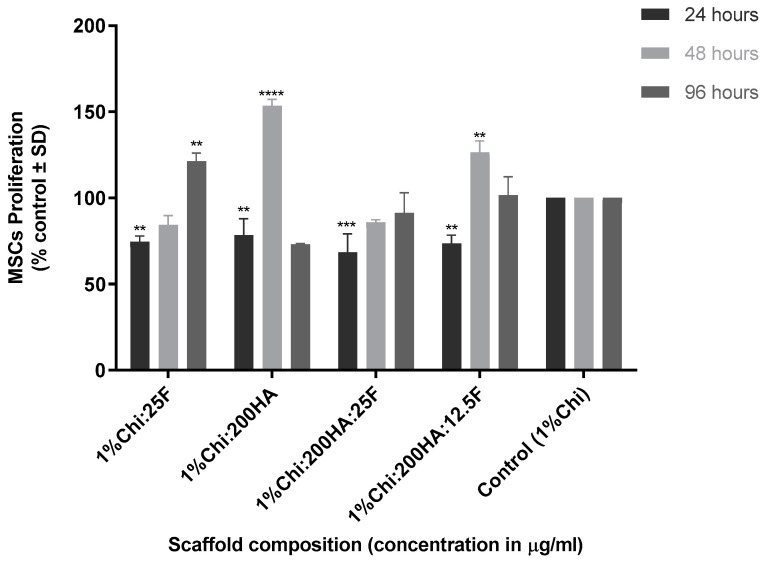
Effect of bi- and tricomposite biomaterials on MSC proliferation. MSCs were seeded onto the bioscaffolds and grown for 96 h, and cell viability was measured using the resazurin assay. The scaffolds are (1% chitosan: 25 µg/mL fucoidan bicomposite; 1%Chi:25F), (1% chitosan:200 µg/mL hydroxyapatite bicomposite; 1%Chi:200HA), (1% chitosan:200 µg/mL hydroxyapatite: 25 µg/mL fucoidan tricomposite; 1%Chi:200HA:25F), (1% chitosan: 200 µg/mL hydroxyapatite: 12.5 µg/mL fucoidan tricomposite; 1%Chi:200HA:12.5F) and (1% chitosan scaffold as a control; 1%Chi). The plates were read at an excitation wavelength of 530 nm and an emission wavelength of 590 nm. The 1% chitosan was treated as a control to calculate % proliferation. Results are expressed as mean ± S.D (*p* < 0.05), *p*-value (* 0.05, ** 0.01, *** 0.001, **** <0.0001), *n* = 3.

Similar to the response of MSCs to fucoidan alone, all composite 3D bioscaffolds had reduced cell numbers compared to the control at 24 h (Figure 7), but this reduction was reversed by 48 h and, in the case of 25 µg/mL of fucoidan and 200 µg/mL of HA, an increase in cell proliferation was observed. These results suggest that the 3D tricomposite is biocompatible, with cell viability being maintained and cell numbers increasing over the next 96 h.

### 2.3. Mesenchymal Stem Cell Differentiation

#### 2.3.1. Alizarin Red Assay

**Figure 8:**
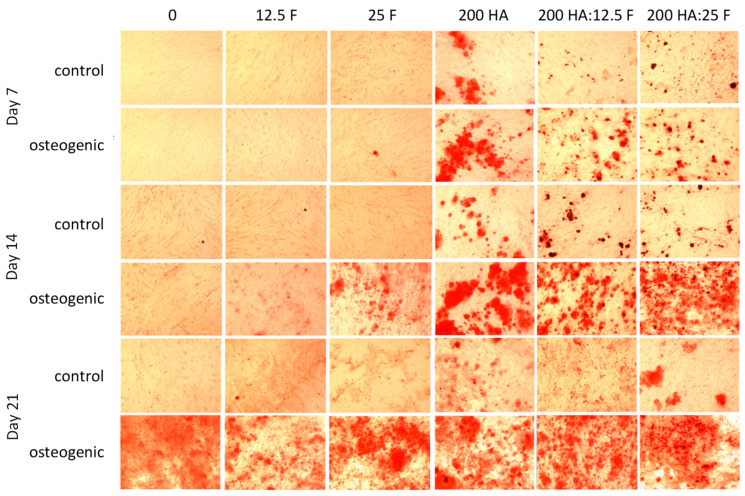
Qualitative evaluation of calcium deposition of differentiating MSCs in the presence of fucoidan, hydroxyapatite and their co-treatment using alizarin red staining. MSCs were cultured with osteogenic supplements and with growth media in the presence of 12.5 and 25 µg/mL of fucoidan (F), 200 µg/mL of hydroxyapatite (HA), 12.5 µg/mL:200 µg/mL and 25 µg/mL:200 µg/mL fucoidan/hydroxyapatite co-treatment, respectively. MSCs cultured with the growth media only were treated as the control. The mineralisation process was monitored over the period of 21 days. (10× original magnification) (*n* = 2).

Alizarin red assay was used to evaluate the mineral calcium deposition of MSCs exposed to fucoidan and HA for up to 21 days (Figure 8). The results showed that MSCs had the potential to differentiate towards an osteogenic lineage, as demonstrated by increasing levels of calcium deposition from day 14. The mineralisation process increased in the presence of fucoidan, 200 µg/mL of HA, and fucoidan/HA co-treatments.

#### 2.3.2. CPC Assay

**Figure 9:**
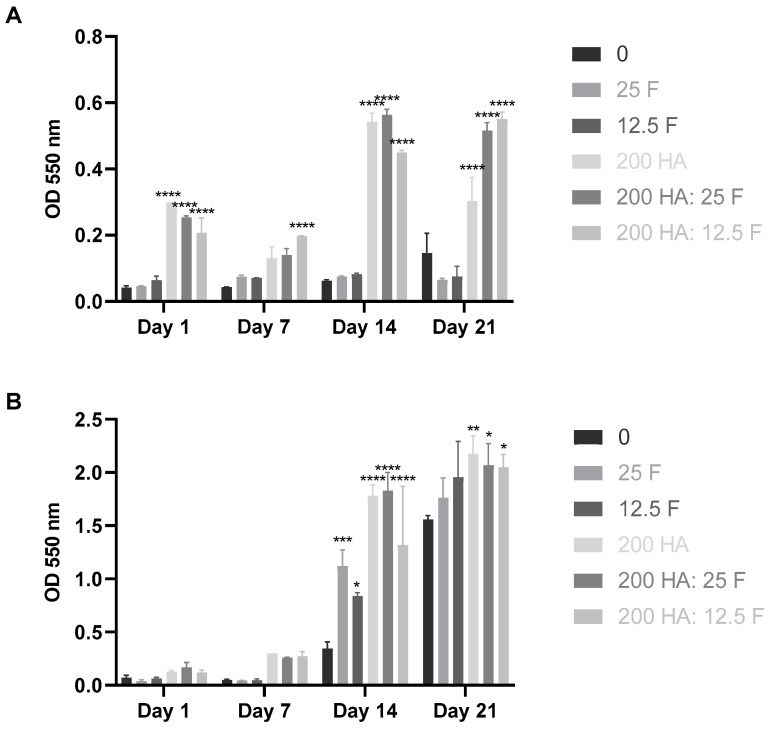
CPC assay for quantitative evaluation of mineralisation of MSCs in growth media without osteogenic supplements (**A**) and osteogenic media (**B**). MSCs cultured on 0, 25 µg/mL, and 12.5 µg/mL of fucoidan (F); 200 µg/mL of hydroxyapatite (HA); and 25 µg/mL: 200 µg/mL and 12.5 µg/mL: 200 µg/mL of fucoidan/hydroxyapatite co-exposure with growth media or osteogenic media over the period of 21 days. The absorbance values on day 1 were subtracted from days 7, 14 and 21 to normalise the quantity of alizarin red (*p* < 0.05). Results are expressed as mean values ± S.D, *p*-value (* 0.05, ** 0.01, *** 0.001, **** < 0.0001), *n* = 3.

Quantitative analysis of staining using CPC assay confirmed a significant increase in mineralisation in the presence of 200 µg/mL of HA and fucoidan/HA co-treatment compared to the control when cells were cultured in the growth media (Figure 9). On day 14, mineralisation in treatments with fucoidan only was also significantly higher than the control when cells were cultured in the osteogenic media (Figure 9B). As expected, osteogenic supplements can boost mineralisation 3–4-fold in comparison to MSCs treated with the growth media only by day 21. The results also showed that MSCs cultured with the control growth media (no osteogenic growth factors) and co-treated with fucoidan and HA demonstrated a 2-fold increase in mineralisation by day 14 compared to MSCs treated with fucoidan and HA individually (Figure 9A).

#### 2.3.3. Alkaline Phosphatase Production

**Figure 10:**
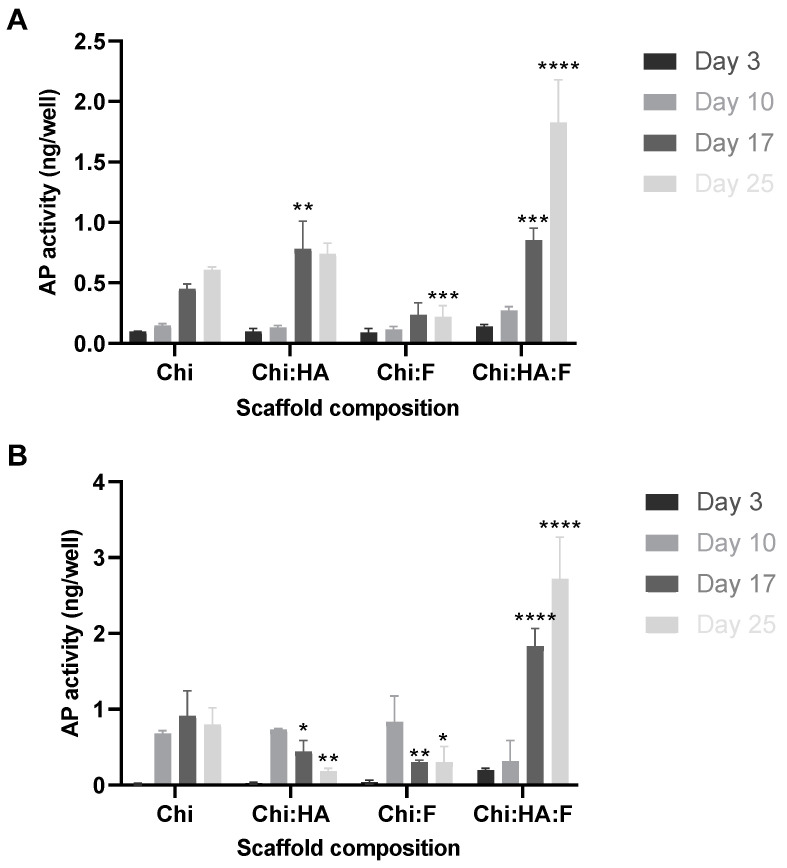
Sensolyte assay for quantitative evaluation of alkaline phosphatase activity in MSCs cultured with growth (**A**) and osteogenic media (**B**) on scaffolds: (1% chitosan; Chi), (1% chitosan with 200 µg/mL hydroxyapatite; Chi:HA), (1% chitosan and 12.5 µg/mL fucoidan; Chi:F) bicomposite and (1% chitosan: 12.5 µg/mL fucoidan: 200 µg/mL hydroxyapatite; Chi:HA:F) tricomposite biomaterial over the period of 25 days. Seeding density of 2.5 × 103 cells per well on 24-well plate. Alkaline phosphatase expression in the presence of chitosan only was taken as the control. Cells at passage less than 6. Results are expressed as mean values ± S.D, *p*-value (* 0.05, ** 0.01, *** 0.001, **** < 0.0001), (*n* = 3).

The impact of single, bi- and tricomposite bioscaffold composition on MSC differentiation towards osteoblasts was evaluated by quantifying MSC alkaline phosphatase activity (in osteogenic and control growth media). When cultured in the control growth media (no osteogenic supplements), a time-dependent increase in alkaline phosphatase production was observed for all treatment conditions from day 3 to day 25, with the exception of the chitosan/fucoidan bicomposite that was found to have significantly reduced alkaline phosphatase production by day 25 (Figure 10A). Addition of fucoidan to the chitosan/HA bicomposite to create the tricomposite increased alkaline phosphatase activity levels by day 17 in comparison to chitosan only, and on day 25, activity levels on the tricomposite was double those induced by the chitosan/HA bicomposite scaffold.

When grown in the presence of osteogenic supplements, the results of the treatment with chitosan only were similar to that seen in the control growth media, with a time-dependent increase in alkaline phosphatase production from days 3 to 25, and, as expected, the levels were higher in the presence of osteogenic supplements (Figure 10B). Both bicomposite formulations decreased alkaline phosphatase activity levels by days 17 and 25. However, the stimulatory effect of the tricomposite was maintained in the presence of osteogenic supplements, with the tricomposite inducing a significant increase in alkaline phosphatase activity by days 17 and 25 (Figure 10B).

**Figure 11:**
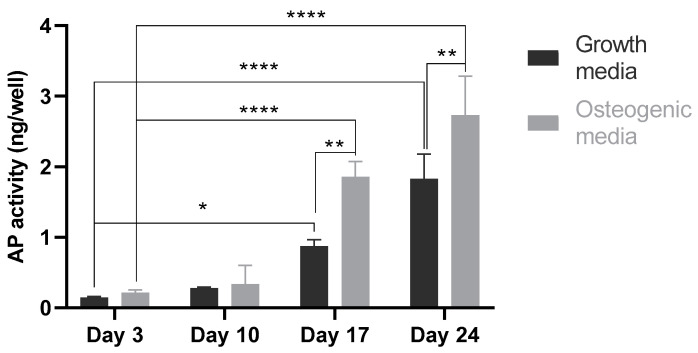
Sensolyte assay for alkaline phosphatase activity in MSCs cultured on tricomposite biomaterial (1% chitosan: 12.5 µg/mL fucoidan: 200 µg/mL hydroxyapatite) over the period of 25 days. Comparison of alkaline phosphatase expression in the presence of osteogenic supplements versus cells grown in the growth media only. Seeding density of 2.5 × 103 cells per well of 24-well plate. Cells at passage less than 6. Results are expressed as mean values ± S.D, *p*-value (* 0.05, ** 0.01, **** < 0.0001), (*n* = 3).

There was a progressive increase in alkaline phosphatase activity from day 3 to day 25 in the presence of both growth and osteogenic media. Relatively higher alkaline phosphatase activity was observed in MSCs supplemented with osteogenic supplements compared to those cultured in the growth media over the period of 25 days. Furthermore, there was no significant difference in alkaline phosphatase levels on day 3 and day 10 between the two treatments (growth and osteogenic media). In contrast, MSCs treated with the osteogenic media showed significantly higher (*p* < 0.001) alkaline phosphatase activity on day 17 and day 25 compared to MSCs cultured with the growth media. The progressive and significant increase in alkaline phosphatase activity from day 0 to days 17 and 25 (*p* < 0.0001) in the presence of the growth media only indicated that the tricomposite had positive impact on osteogenic differentiation (Figure 10B and Figure 11).

## 3. Discussion

Chitosan, an amino polysaccharide that is a deacetylated derivative of chitin, is widely used for bone tissue engineering due to its good biocompatibility, biodegradability, osteoconductivity and antibacterial properties [54,55]. Chitosan can be easily combined with organic or inorganic molecules to improve its mechanical properties and accelerate bone formation. To date, many different compounds, including ceramics and polymers, have been used in combination with chitosan to form bone regeneration scaffolds [55,56,57,58]. In the study by Zafeiris et al. [58], chitosan was combined with hydroxyapatite and chemically crosslinked using genipin.

The porosity and mechanical properties of the obtained scaffold were similar to those that characterised cancellous bone. The biocompatibility analysis revealed that the scaffold allowed cell adherence and maintained their high viability. Hydroxyapatite, a calcium phosphate cement type, is commonly used in combination with chitosan to create bone regeneration scaffolds due to the biocompatibility, osteoconductivity and biodegradation of this hybrid material [59,60,61,62]. Lu et al. [63] developed hydroxypropyl chitosan-based scaffold with incorporated nano-hydroxyapatite and marine-derived polysaccharide, namely fucoidan. Used by researchers, hydroxypropyl chitosan is a semisynthetic chitosan derivative with known antibacterial and antioxidant activities. However, this material is characterised by its hydrophilic nature, low crystallinity and poor mechanical resistance. To overcome that, tested scaffolds were developed by incorporating nano-hydroxyapatite into hydroxypropyl chitosan-based matrix and crosslinking using genipin. Fucoidan, a marine-derived polysaccharide with osteoconductive and osteogenic activities, was adsorbed to these scaffolds via electrostatic interactions. The addition of nanohydroxyapatite and fucoidan improved osteoblast mineralisation, which resulted in increased alkaline phosphatase level.

The aim of our study was to develop and evaluate a tricomposite biomaterial composed of chitosan, fucoidan and hydroxyapatite for bone regeneration. Firstly, the optimum ratio of each component was determined by conducting cytotoxicity and cell differentiation studies with varying concentrations of these individual components. Human MSCs were exposed to 0 to 1000 µg/mL of fucoidan and hydroxyapatite. Fucoidan revealed a dose-dependent effect on MSC proliferation, with cytotoxicity being demonstrated in cells treated with concentrations higher than 25 µg/mL, and a slight increase (albeit non-significant) in cell proliferation when treated with 25 µg/mL to 6.25 µg/mL of fucoidan for 72 h. Similarly, when exposed to HA, there was a trend towards increased cell proliferation at 200 µg/mL, but significant cytotoxicity was observed at the highest dose of 1000 µg/mL. Based on these results, concentrations of 25 mg/mL and 12.5 mg/mL of fucoidan and 200 mg/mL of HA were chosen for continued development of composite chitosan scaffolds. The biocompatibility and osteogenic properties of these components were confirmed when fucoidan and hydroxyapatite were given in combination. Both concentrations (25:200 µg/mL and 12.5:200 µg/mL) had minimal effects on MSC proliferation but were found to boost osteogenic differentiation with increased calcium deposition and mineralisation by days 7, 14 and 21.

A tricomposite scaffold was then developed using a modified poly-blend method [33]. The SEM images revealed a uniform distribution of hydroxyapatite nanoparticles and a uniform pore size throughout the scaffold. The crystal size of hydroxyapatite is crucial for the activity of osteoblasts and scaffold viability [64,65]. In addition, fucoidan was clearly evident as thread-like structures integrated and projecting from the scaffold surface, which was most encouraging as it would be readily available and accessible to cells. The FTIR analysis presented the overall elemental composition of the scaffold, which confirmed the presence of each of the constituent biomaterials in the tricomposite with prominent calcium, phosphate and sulphur peaks, all being important in scaffolds for bone tissue engineering.

Pore microstructure is an important factor in any scaffold construction. The key characteristics include pore size, inter-connection between pores, porosity and surface-to-volume ratio. The ideal scaffold should be highly porous with a proper pore size to support cell migration, cell proliferation and vascularisation deep inside the pores, and permeable to facilitate the ingrowths of blood vessels, the transportation of nutrients and the removal of waste products [66,67,68,69]. The SEM results showed that the tricomposite scaffold has a highly porous structure with uniform pore distribution throughout the scaffold. This was confirmed by measuring scaffold porosity and density using a liquid displacement method with ethanol, which found that the incorporation of 200 µg/mL of HA into the scaffold resulted in an increased scaffold density. The porosity studies showed that the tricomposite scaffold had >90% porosity. This is important as it promotes vascularisation and facilitates osteoblast proliferation and differentiation. In addition, a porous surface improves mechanical stability and interlocking at the critical interface between the biomaterial and surrounding natural bone [70]. Although porosity was reduced in the tricomposite compared to the scaffold with chitosan alone, this was accompanied by an increase in scaffold density. A higher density of the scaffold leads to greater mechanical strength and, thus, there is a trade-off between strength and a porosity high enough to provide a favourable biological environment. As the addition of negatively charged fucoidan increased the availability of free functional groups in the chitosan composite system, it was expected that the chitosan/fucoidan bicomposite would have a greater swelling ratio and that this would increase with time. This was confirmed, but the increase in ratio was reduced when fucoidan was contained in a tricomposite scaffold. The absorption properties of chitosan/HA and chitosan/HA/fucoidan scaffolds were less efficient in comparison to the chitosan/fucoidan scaffold because of the presence of the HA component [71].

Cytotoxicity analysis was performed to determine the biocompatibility of the scaffolds for bone tissue engineering applications. Cytotoxicity and differentiation studies of MSCs on the chitosan bi- and tricomposites showed that there was some evidence of a stimulatory effect on cell proliferation and very strong evidence that the tricomposite scaffold enhanced osteogenic differentiation in comparison to that seen with the inclusion of the individual components. The quantitative analysis of staining using CPC assay showed that MSCs treated with fucoidan and HA in the growth media demonstrated increase in mineralisation by day 14 in comparison to the individual treatments. Hydroxyapatite and fucoidan induced mineralisation process in the tested cell line. Improved osteoconductivity and MSC proliferation were observed for the hydroxyapatite-and-chitosan scaffolds [72]. The ability of fucoidan to induce mineralisation in osteoblasts, MSCs and iPS was reported in previous studies as well [48,73,74].

The activity of alkaline phosphatase, a biochemical marker of osteoblast phenotype, increased almost two-fold in MSCs cultured on the chitosan/HA/fucoidan tricomposite [39,75,76,77,78]. The ability of fucoidan to significantly increase the expression of alkaline phosphatase and osteocalcin, markers for osteogenesis, was demonstrated in previous studies [45,46,47,79]. According to Kim et al. [79], the observed fucoidan effect on osteoblast differentiation, such as significantly increased alkaline phosphatase activity, calcium accumulation and expression of osteoblast-specific genes, was mediated through BMP2–Smad 1/5/8 signalling by activating ERK and JNK. Fucoidan was found to have higher affinity for BMP-2 binding than heparin [80]. Hydroxyapatite/fucoidan bio-nanocomposites were found to stimulate bone formation through increased osteoblastic activity [81]. Moreover, several studies showed that incorporation of fucoidan into hydroxyapatite/chitosan/alginate scaffolds made them more effective in comparison to scaffolds without this marine polysaccharide [82,83,84].

The bone-inducing property of a tricomposite biomaterial composed of chitosan, fucoidan and hydroxyapatite was evaluated in the present study using bone marrow-derived human mesenchymal stem cells. The developed scaffold is biocompatible and promotes osteogenesis, and its structural and compositional properties meet the requirements of bone tissue engineering scaffolds. Further studies can be conducted to examine scaffold degradation and resorption and actual bone formation. Scaffold degradation/resorption is an important factor in bone tissue engineering. To facilitate bone repair and to allow new bone to fully restore the native tissue, a scaffold should degrade over time. Both too rapid and too slow rate of degradation may result in failures during the bone regeneration process [85,86].

## 4. Materials and Methods

### 4.1. Biomaterial Synthesis

#### 4.1.1. Chitosan Scaffold Preparation

Using medium-molecular-weight chitosan (Sigma Aldrich, Arklow, Ireland), a 1% solution was prepared by adding 1 g of chitosan powder to 0.05% acetic acid solution (Sigma Aldrich, Arklow, Ireland). The solution was stirred at 50 °C until completely dissolved. For precipitation, 1M NaOH (AnalaR NORPUR, Leuven, Belgium) was added drop wise until the pH reached 8. Following centrifugation at 10,000× *g* for 15 min, the supernatant was discarded, and the remaining gel was washed with water to remove salts. The process of dissolution in 0.05% acetic acid solution, precipitation, centrifugation and washing was repeated several times until no further change in colour was observed.

For scaffold preparation, 1% chitosan solution was prepared in 0.05% acetic acid using the purified chitosan obtained above and neutralised by adding 1 M NaOH. Then, 1 mL of the 1% chitosan solution was poured into each well of a 24-well plate, which had an average depth of 5 mm, and stored at −80 °C to prepare for freeze drying. Freeze drying was performed using the Freezone^®^ 2.5 freeze dry system (Labconco, Kansas City, MO, USA) at −50 °C and a vacuum of 133 × 10^−3^ mbar for 24 h. The scaffolds were sterilised with 70% ethanol, followed by three rinses in sterile PBS and an overnight wash in PBS. Before seeding cells, the scaffolds were placed in a complete growth medium and incubated at 37 °C for 2 h.

#### 4.1.2. Fucoidan Preparation

Fucoidan (*Fucus vesiculosus,* Sigma Aldrich, Arklow, Ireland) was prepared as a 10 mg/mL stock solution in PBS, filtered using 0.2 μm filter and sterilised via autoclaving. The effect of autoclaving on fucoidan was evaluated by comparing the fast-performance liquid chromatography (FPLC) spectral profile with the profile of a non-autoclaved fucoidan sample (results not shown) which confirmed that autoclaving did not have any detrimental effect. For cell culture studies, 200, 100, 50, 25, 12.5, 6.25 and 3.125 μg/mL solutions were prepared from the stock solution using a complete growth medium.

#### 4.1.3. Hydroxyapatite

HA (nano-powder < 200 nm particle size, Sigma Aldrich, Arklow, Ireland) was prepared as a 10 mg/mL stock solution in PBS and autoclaved. The stock solution was then used to prepare 200, 100, 50, 25, 12.5, 6.25 and 3.125 μg/mL using a complete growth medium.

#### 4.1.4. Bicomposite Scaffold Preparation

Chitosan solution (1%) was prepared in 0.05% acetic acid using the purified chitosan as above. pH was adjusted to the upper maximum limit of 6.86, and then either the HA stock solution (10 mg/mL) was added to a final concentration of 200 μg/mL or the fucoidan solution (10 mg/mL) was added to give final concentrations of 12.5 μg/mL and 6.25 μg/mL. To ensure uniform dispersion, the solutions were stirred at 50 °C in a sonication bath for 2 h. Then, 1 ml of each solution was aliquoted into a 24-well plate as required and freeze dried as before.

#### 4.1.5. Tricomposite Scaffold Preparation

The tricomposite scaffolds were prepared using a poly-blend method adapted from Thein-Han et al. [33]. In this process, 1% of the purified chitosan solution was prepared, followed by addition of fucoidan to give a final concentration of 12.5 μg/mL. This solution was mixed for 1 h using a magnetic stirrer, followed by drop-by-drop addition of the HA stock solution to a final concentration of 200 μg/mL. This addition was carried out whilst the chitosan/fucoidan solution was being stirred and was followed by a 2 h sonication step. A total of 1 mL of tricomposite solution was transferred to a 24-well plate, frozen at −80 °C and subsequently freeze-dried. The scaffolds were sterilised using 70% ethanol and washed in PBS as before.

### 4.2. Scaffold Characterisation

#### 4.2.1. Scanning Electron Microscopy (SEM)

The microstructure of the scaffolds was examined via SEM. The samples were sputter-coated with gold and observed using a scanning electron microscope. Imaging was carried out using a TableTop SEM (Hitachi High-Technologies Corp., Tokyo, Japan) operated at 15 kV at 100×, 1000× and 5000× magnification power.

#### 4.2.2. Fourier-Transform Infrared Spectroscopy (FTIR)

Spectra were collected to analyse and compare the material characteristics of all scaffold samples, specifically CH only, CH:HA bicomposite and CH:HA:FU tricomposite. The FTIR analysis was carried out using a Spectrum One FTIR (Perkin Elmer, Beaconsfield, UK). The scaffolds were finely cut and mixed with potassium bromide before being pressed and analysed. The FTIR spectra were collected with wavenumbers between 4000 and 400 cm^−1^. The scans were evaluated to determine the major elements present and to detect the presence of HA and FU in the bi- and tricomposite scaffolds.

#### 4.2.3. Porosity and Density

Porosity and density of the scaffolds were measured using a liquid displacement method with ethanol [87]. Briefly, the starting volume of ethanol was recorded along with the dry weight of the scaffolds (W). Following immersion of the scaffolds in ethanol for 48 h to saturate, the residual ethanol volume was recorded. The following parameters were used:

*V*1 = Initial known volume of ethanol

*V*2 = Total volume sum of scaffold and ethanol

*V*3 = Volume of ethanol after scaffold is removed

The volume of each scaffold was calculated as *V*2 − *V*1 and total volume of the scaffold was then estimated as *V* = *V*2 − *V*3. Porosity was calculated as follows:

$$Porosity=\frac{v1-v3}{v2-v3}$$

Apparent density was calculated as follows:

$$Density=\frac{w}{v2-v3}$$

Finally, water retention property/swelling ratio was calculated. The scaffolds were weighed before and after immersion in distilled H_2_O for 24, 48 and 72 h. The swelling ratio was calculated as follows [14]:

$$\mathrm{welling}\mathrm{ratio}=\frac{\mathrm{wet}\mathrm{weight}-\mathrm{dry}\mathrm{weight}}{\mathrm{dry}\mathrm{weight}}$$

### 4.3. In Vitro Analysis

#### 4.3.1. Cell Culture

The primary cell source was bone marrow-derived human mesenchymal stem cells (MSCs), which were obtained from the vertebral body, following ethical approval and informed consent, of a 36-year-old and a 33-year-old male during intra-vertebral pedicle screw implantation. MSCs were isolated from the bone marrow samples as previously described [88]. MSCs were cultured in Alpha MEM Glutamax media (Gibco^®^, Thermo Fisher Scientific, Waltham, MA, USA), supplemented with 10% HyClone™ Defined Fetal Bovine Serum (Thermo Fisher Scientific, Waltham, MA, USA) and 1% Penicillin (Gibco^®^, Life Technologies) at 37 °C and 5% CO_2_. The media were changed every two days until these cells reached a confluency of 80–90%. Cells were used no later than passage 6.

#### 4.3.2. Biocompatibility

Cytotoxicity of the single, bi- and tricomposites to MSCs was determined. For fucoidan and HA alone, 1 × 10^4^ MSCs were seeded into a 96-well plate and, following an attachment period, cells were exposed to a solution of 1000, 200, 100, 50, 25, 12.5, 6.25 or 3.125 μg/mL. Cytotoxicity of the scaffold with chitosan alone or when combined into bi- and tricomposites was evaluated by seeding 2.5 × 10^3^ MSCs onto each scaffold on a 24-well plate, i.e., scaffold containing either 1% chitosan, 1% chitosan/200 μg/mL HA, 1% chitosan/2.5 μg/mL fucoidan, or 1% chitosan/200 μg/mL HA/12.5 μg/mL fucoidan. Cellular respiration was evaluated using resazurin assay (TOX 8, Sigma Aldrich) at 24, 48 and 72 h, according to the manufacturer’s instructions. Resazurin solution (1 mg/mL) was added to the cells in an amount equal to 10% of the culture medium (*v/v*) and incubated at 37 °C under 5% CO_2_ for 3 h. Fluorescence was detected at an excitation wavelength of 530 nm and an emission wavelength of 590 nm.

#### 4.3.3. Mesenchymal Stem Cell Differentiation

##### Alizarin Red

Osteogenic differentiation following exposure to fucoidan and HA was determined using alizarin red assay. Coverslips coated with 0.1% gelatine were placed in 24-well plates and seeded with 2.5 × 10^3^ MSCs, followed by exposure to either the growth media or osteogenic media (αMEM, 10% FBS, 50 μM ascorbate-2-phosphate, 0.1 μM dexamethasone, 10 μM β-glycerophosphate and 1% pen/strep) supplemented with 12.5 or 25 µg/mL fucoidan, 200 µg/mL HA, 12.5 µg/mL fucoidan:200 µg/mL HA, or 25 µg/mL fucoidan:200 µg/mL HA for up to 21 days. MSCs cultured with the growth media alone served as the negative control.

A 2% solution of Alizarin Red S was prepared and adjusted to pH of 4.1–4.3. Cells were washed with PBS without disrupting the cell monolayer and fixed in neutral buffered formalin (10%) at RT for 30 min. After washing with distilled water, 1 mL of Alizarin Red S was added and incubated in the dark at room temperature for 45 min. Following additional washing, the coverslips were transferred to glass slides and mounted with DPX for qualitative light microscopy. In a duplicate experiment to quantify the staining, 1 mL of 10% cetylpyridium chloride solution (Sigma Aldrich) was added to each well and incubated for 20 min at 37 °C to elute the stain. Then, 200 μL of this eluted stain was added to 96-well plates and read at an absorbance wavelength of 550 nm using a spectrophotometer (Varioskan, Thermo Fisher Scientific, Waltham, MA, USA).

##### Alkaline Phosphatase

Osteogenic differentiation following exposure of cells to chitosan single, bi- and tricomposites was determined using an alkaline phosphatase activity assay. As before, 2.5 × 10^3^ MSCs were seeded onto each scaffold of 1% chitosan, 1% chitosan/200 µg/mL HA, 1% chitosan/12.5 µg/mL fucoidan or 1% chitosan/12.5 µg/mL fucoidan/200 µg/mL HA on a 24-well plate and cultured in either the growth medium or osteogenic medium for periods up to 25 days. At each time point, the cells were washed with 1X assay buffer and then lysed in 0.002% Triton X-100 assay buffer with agitation at 4 °C for 10 min. Following centrifugation at 2500× *g* for 10 min at 4 °C, the supernatant was collected and used in the Sensolyte^®^ pNPP Alkaline Phosphatase Assay Kit (AnaSpec Inc, Fremont, CA, USA) for quantitative evaluation of alkaline phosphatase production according to the manufacturer’s instructions. Absorbance was read at 405 nm.

### 4.4. Statistical Analysis

Two-way ANOVA followed by Tukey’s multiple comparison tests was performed using GraphPad Prism version 9 for Windows, GraphPad Software, La Jolla, CA, USA. *p* < 0.05 was considered to be significant.

## 5. Conclusions

In this study, a highly porous tricomposite scaffold with a uniform distribution of hydroxyapatite nanoparticles and fucoidan was successfully developed using a poly-blend method. The optimum ratio of each component required for effective osteoinduction and biocompatibility and the osteoinductive capacity of the tricomposite were determined. It was found that incorporation of fucoidan into the scaffold composition could enhance the water absorption properties. This behaviour was specifically observed in the fucoidan bicomposite with chitosan. The tricomposite scaffold containing chitosan, fucoidan and hydroxyapatite showed an elevated level of alkaline phosphatase activity. The porosity study revealed that the tricomposite scaffold has a porous structure of over 90% porosity, and a relatively higher density was achieved in the developed tricomposite scaffold compared to the chitosan scaffold. The cytotoxicity and differentiation studies of MSCs on the bi- and tricomposites showed some evidence of a stimulatory effect on cell proliferation and very strong evidence that the tricomposite scaffold enhances osteogenic differentiation more than that seen with the inclusion of the individual components. By considering all of these factors, it can be anticipated that the developed tricomposite scaffold will mimic bone extracellular matrix by promoting stem cell and osteo-progenitor cell differentiation into osteoblasts, which, in turn, will lead to a functional bone matrix that correlates with in vivo bone formation, regeneration and repair.

It can be concluded that the tricomposite is biocompatible, promotes osteogenesis, and has the structural and compositional properties required of a scaffold for bone tissue engineering.

## Figures and Tables

**Table 1:** Porosity, density and swelling ratio measurements of the bioscaffolds. The evaluated scaffolds are chitosan (Chi; 1%), chitosan/hydroxyapatite (Chi:HA; 1%:200 µg/mL), chitosan/fucoidan (Chi:F; 1%:12.5 µg/mL) and chitosan/fucoidan/hydroxyapatite (Chi:HA:F; 1%:12.5 µg/mL:200 µg/mL) tricomposite. (*n* = 3).

	Scaffold Composition			
Parameter	Chi	Chi:HA	Chi:F	Chi:HA:F
Porosity [%]	93.75 ± 7.03	77.59 ± 5.82	89.74 ± 6.73	92.05 ± 6.90
Density [g/cm^3^]	0.03 ± 0.002	0.05 ± 0.003	0.04 ± 0.003	0.05 ± 0.003
Swelling ratio				
2 h	6.39 ± 0.32	5.49 ± 0.27	5.72 ± 0.29	4.86 ± 0.24
48 h	5.74 ± 0.29	5.68 ± 0.28	6.99 ± 0.35	5.71 ± 0.29
96 h	6.16 ± 0.31	6.25 ± 0.31	8.28 ± 0.41	5.94 ± 0.30

## Data Availability

Data are contained within the article.
